# Luteolin Alleviates Methamphetamine-Induced Hepatotoxicity by Suppressing the p53 Pathway-Mediated Apoptosis, Autophagy, and Inflammation in Rats

**DOI:** 10.3389/fphar.2021.641917

**Published:** 2021-02-19

**Authors:** Kai-Kai Zhang, Hui Wang, Dong Qu, Li-Jian Chen, Li-Bin Wang, Jia-Hao Li, Jia-Li Liu, Ling-Ling Xu, Jamie Still Yoshida, Jing-Tao Xu, Xiao-Li Xie, Dong-Ri Li

**Affiliations:** ^1^ Department of Forensic Pathology, School of Forensic Medicine, Southern Medical University, Guangzhou, China; ^2^ Department of Pediatric Surgery, Guangzhou Institute of Pediatrics, Guangzhou Women and Children’s Medical Center, Guangzhou Medical University, Guangzhou, China; ^3^ Department of Toxicology, School of Public Health, Southern Medical University, Guangzhou, China; ^4^ Faculty of Health Sciences, Butsuryo College of Osaka, Sakai, Japan; ^5^ Shanghai Key Laboratory of Forensic Medicine, Institute of Forensic Science, Ministry of Justice, Shanghai, China; ^6^ Department of Forensic Clinical Medicine, School of Forensic Medicine, Southern Medical University, Guangzhou, China; ^7^ Department of Forensic Evidence Science, School of Forensic Medicine, Southern Medical University, Guangzhou, China

**Keywords:** methamphetamine, luteolin, hepatotoxicity, protective effect, p53 signaling pathway

## Abstract

Misuse of the psychostimulant methamphetamine (METH) could induce serious hepatotoxicity. Our previous study revealed the effects of luteolin on alleviating METH-induced hepatotoxicity, however, the detailed mechanisms have not been elucidated. In this study, rats were orally pretreated with 100 mg/kg luteolin or sodium dodecyl sulfate water, and then METH (15 mg/kg, intraperitoneal [i.p.]) or saline was administered. Histopathological and biochemical analyses were used to determine the alleviative effects of luteolin. Based on the RNA-sequencing data, METH induced 1859 differentially expressed genes (DEGs) in comparison with the control group, which were enriched into 11 signaling pathways. Among these DEGs, 497 DEGs could be regulated through luteolin treatment and enriched into 16 pathways. The p53 signaling pathway was enriched in both METH administered and luteolin pretreated rats. Meanwhile, luteolin significantly suppressed METH-induced elevation of p53, caspase9, caspase3, cleaved caspase3, the ratio of Bax/Beclin-2, as well as autophagy-related Beclin-1, Atg5, and LC3-II. Luteolin also relieved METH-induced hepatotoxicity by decreasing inflammation factors, including TNF-α, IL-1β, and IL-18. Moreover, the levels of PI3K, *p*-Akt, and the normalized ratio of *p*-Akt/Akt declined after METH administration, whereas luteolin pretreatment failed to reverse these effects. Our results suggest that luteolin alleviates METH-induced hepatic apoptosis, autophagy, and inflammation through repressing the p53 pathway. It further illustrates the protective mechanisms of luteolin on METH-induced hepatotoxicity and provides a research basis for clinical treatment.

## Introduction

Methamphetamine (METH), a highly addictive stimulant, has become a public health problem due to its abuse globally (Centazzo et al., 2019; Xu et al., 2019; Xu and Liu, 2019). METH could cause abnormal behavioral phenotypes and result in neurotoxicity, including mediating oxidative stress, promoting neuroinflammation, stimulating neural apoptosis, and autophagy (Yamamoto and Bankson, 2005; Park et al., 2017; Tan et al., 2020). Increasing evidence shows that METH could also cause multiple organs damage, including the liver (Qu et al., 2020). Complex mechanisms are involved in METH-induced hepatotoxicity, including mediating hepatic metabolic disorders, stimulating oxidative stress, promoting hyperthermia, and inducing mitochondrial impairment (Willson, 2019). Our previous study confirmed that METH-induced hepatic injury was related to the blocking of multiple cellular processes, such as cell division and cycle, which might accelerate hepatic apoptosis (Wang et al., 2017). However, there are limited effective treatments for METH-induced hepatotoxicity.

Luteolin (3,4,5,7-tetrahydroxy flavone), a type of flavonoid, is generally found richly in natural vegetables, fruits, and other plants (Seelinger et al., 2008). Luteolin is extensively utilized to treat multiple diseases in Chinese traditional medicine including tumor, allergy, oxidation, inflammation, apoptosis, and autophagy (Manzoor et al., 2019; Tan et al., 2020). Also, luteolin has been reported to alleviate multiple hepatic injuries. Luteolin protects against galactosamine/lipopolysaccharide-induced hepatic apoptosis, inflammation, and oxidative stress (Lee et al., 2011; Park and Song, 2019). Mercuric chloride-induced hepatotoxicity can also be ameliorated by luteolin through modulating the Nrf2/NF-κB/p53 signaling pathway (Zhang et al., 2017). Our previous study showed that luteolin effectively alleviated METH-induced hepatotoxicity. Multiple pathways could contribute to its protective effects, though the detailed mechanisms haven’t been clarified (Qu et al., 2020).

The p53 signaling pathway has been recognized as a crucial regulator of multiple biological processes, including tumor growth, cell cycle arrest, DNA repair, cell metabolism, necrosis, and proliferation (Aubrey et al., 2018). The p53 signaling pathway is also a regulator of apoptosis and autophagy (Robin et al., 2019). Meanwhile, p53 shows a close association with PI3K/Akt signaling pathway (Grinkevich et al., 2017), and our previous study has confirmed its key role in luteolin’s protective effects on METH-induced neurotoxicity (Tan et al., 2020). This study examined whether this pathway also participates in the hepatic protection by luteolin.

Rats were pretreated with luteolin, followed by the administration of METH. Histopathological and biochemical analyses were performed to determine the hepatic damages. The potential pathways involved in the protective effects of luteolin were enriched and the detailed mechanisms were investigated based on RNA-sequencing. These findings may lead to the development of therapeutic drugs for METH-induced hepatotoxicity.

## Materials and Methods

### Chemicals and Drugs

METH was obtained from the National Institute for the Control of Pharmaceutical and Biological Products (Beijing, China) and the purity was >99%. The purity of luteolin was >96% (Push Bio-Technology Co., Ltd. (Chengdu, China)), whereas 0.5% Sodium dodecyl sulfate (SDS) water was utilized as its solvent.

### Animals and Treatments

Sprague Dawley (SD) rats (6–8 weeks old, male, 200 ± 5 g) were purchased from the Experimental Animal Center of Southern Medical University. All rats were allowed to acclimatize in the SPF animal room for 1 week before the experiment (light/dark cycle, 12 h; room temperature, 22 ± 2°C). Food and water were freely available during this period. All experimental steps strictly followed the National Institute of Health Guide for the Care and Use of Laboratory Animals of the Southern Medical University. The number of the Ethical Committee Approval Code was L2018123.

**FIGURE 1:**
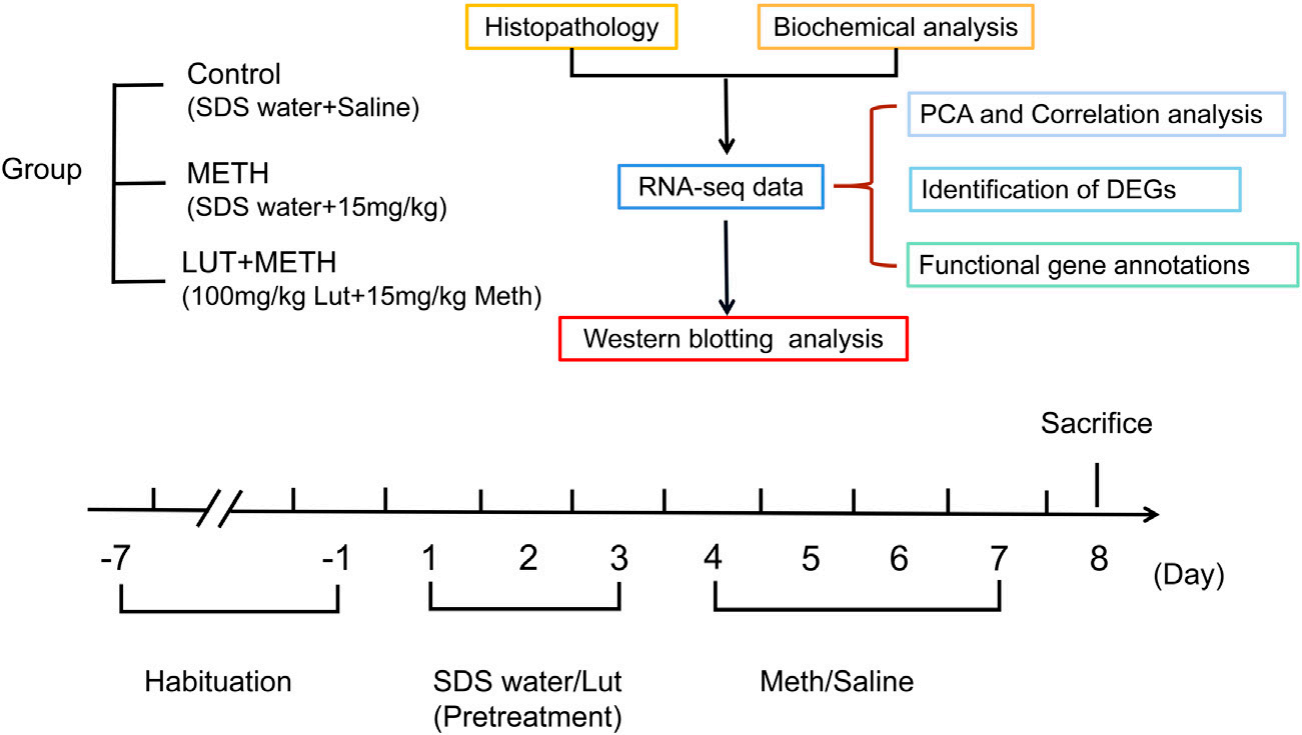
Experimental design for METH exposure and luteolin pretreatment.

All the rats were randomly assigned to three groups each having six rats: the Control, METH, and luteolin pretreated group (Figure 1) (Qu et al., 2020; Tan et al., 2020). In brief, rats were orally pretreated with luteolin (100 mg/kg) or 0.5% SDS water (once daily) for 3 days. Subsequently, 15 mg/kg METH or an equal volume of saline (i.p.) were intraperitoneally (i.p.) injected at 12 h intervals for four consecutive days. All rats were sacrificed under deep anesthesia (60 mg/kg i. p. pentobarbital sodium)12 h after the final injection (Xu et al., 2018).

Blood samples were centrifuged (3,000 r/mins, 10 min s) at room temperature to separate plasma, and then stored at −80°C for biochemical analysis. One-half of the liver tissues were fixed with 10% phosphate-buffered formalin, whereas the other tissues were rapidly frozen in liquid nitrogen and stored at −80°C for further detection.

### Histopathology

After fixing for 24 h, liver tissues were embedded in paraffin and sectioned at a 3 μm thickness on a manual rotary microtome. Hematoxylin and eosin (HandE) staining was conducted for further histopathological examination (Xie et al., 2019).

### Biochemical Analysis

An Enzyme-linked immunosorbent assay (CUSABIO Biotechnology, Wuhan, China) was performed to examine the degree of liver damage and evaluate the protective effects of luteolin. This was to determine the levels of aspartate transaminase (AST) and alanine aminotransferase (ALT) in plasma.

### RNA-Seq Data Processing, Analysis and Result Reporting

#### Principal Component Analysis and Correlation Analysis of Samples

Based on our published RNA-seq data (Qu et al., 2020) [BioProject: PRJNA529763], principal component analysis (PCA) was performed to integrate the principal component and simplify the complexity of the sample reads (http://deweylab.biostat.wisc.edu/rsem/) (Giuliani, 2017). The high similarity samples and the outliers were filtered out according to the relationship and the size of variation among samples.

Similarly, Correlation analysis was conducted to determine the variation of repeat samples and evaluate the reliability of experimental treatment.

#### Identification of Differentially Expressed Genes

Fragments per Kilobases per Million reads (FPKM) method was performed to avoid the influence of length difference and compare the expression level of genes (Li and Dewey, 2011). The DESeq2 tool was utilized to assess the differential genes to accurately screen out DEGs. The screening parameters were set as: the fold change ≥2 or ≤0.5, *p*-value < 0.05.

### Functional Gene Annotations

The Kyoto Encyclopedia of Genes and Genomes (KEGG) analysis (https://www.genome.jp/kegg/) and Protein-Protein Interaction (PPI) tools (https://string-db. org/and Cytoscape_v3.6.1) were conducted for enrichment analysis and protein interactions respectively to analyze the potential function of the DEGs (Kanehisa et al., 2017; Szklarczyk et al., 2017). Annotations of the DEGs referred to the DAVID (http://david.ncifcrf.gov) and the STRING database (http://string.db.org).

### Western Blotting Analysis

The isolated total protein was separated using SDS-PAGE gels (20 ug, per sample) and transferred to polyvinylidene difluoride (PVDF, 0.22uM) membranes (Qu et al., 2019; Zhao et al., 2020). The following steps were then conducted on the membranes orderly: Blocked in 5% skim milk for 2 h at room temperature, incubated with the primary antibodies overnight at 4°C and the secondary antibodies for 1 h at room temperature. Finally, the blots were visualized on the ECL System. The following primary antibodies were utilized: Anti-P53 (diluted 1:1,000; 4 A Biotech), anti-caspase9 (diluted 1:1,000, Proteintech), anti-caspase3 (diluted 1:1,000; ABclonal), anti-cleaved caspase3 (diluted 1:1,000; CST), anti-Bax (diluted 1:1,000; CST), anti-Beclin2 (diluted 1:1,000; Proteintech), anti-Beclin1 (diluted 1:1,000; CST), anti-ATG5 (diluted 1:1,000; HuaAn Biotechnology), anti-LC3 (diluted 1:1,000; Proteintech), anti-TNF-α (diluted 1:1,000, Proteintech), anti-IL-1β (diluted 1:1,000, Proteintech), anti-IL-18 (diluted 1:1,000, Proteintech), anti-PI3K p85 alpha(diluted 1:1,000; Proteintech), anti-AKT (diluted 1:1,000; Proteintech), anti-AKT-phospho-S473 (diluted 1:1,000; Proteintech) and anti-Beta-Actin (diluted 1:1,000; 4 A Biotech). The secondary antibodies (HRP-labeled goat anti-mouse IgG (H + L) or anti-rabbit IgG (H + L), Beijing Dingguo Changsheng Biotechnology) were diluted at the concentration of 1:5,000.

### Statistical Analysis

All biochemical analysis and Western Blotting experiments were carried out in triplicate. All values were reported as mean ± SEM. The GraphPad Prism version 6.0 (San Diego, United States) was utilized for statistical analysis. One-way analysis of variance (ANOVA) followed by post-hoc Tukey tests, was used for comparisons of multiple groups. PCA analysis was performed on RSEM software (version 1.3.1) with TPM methods and the Pearson correlation analysis was employed to analyze the correlation among samples, while DESeq2 tool (version 1.24.0) was utilized to screen the DEGs. Values of *p* < 0.05 were considered statistically significant.

## Results

### Luteolin Effectively Alleviated METH-Induced Hepatotoxicity

**FIGURE 2:**
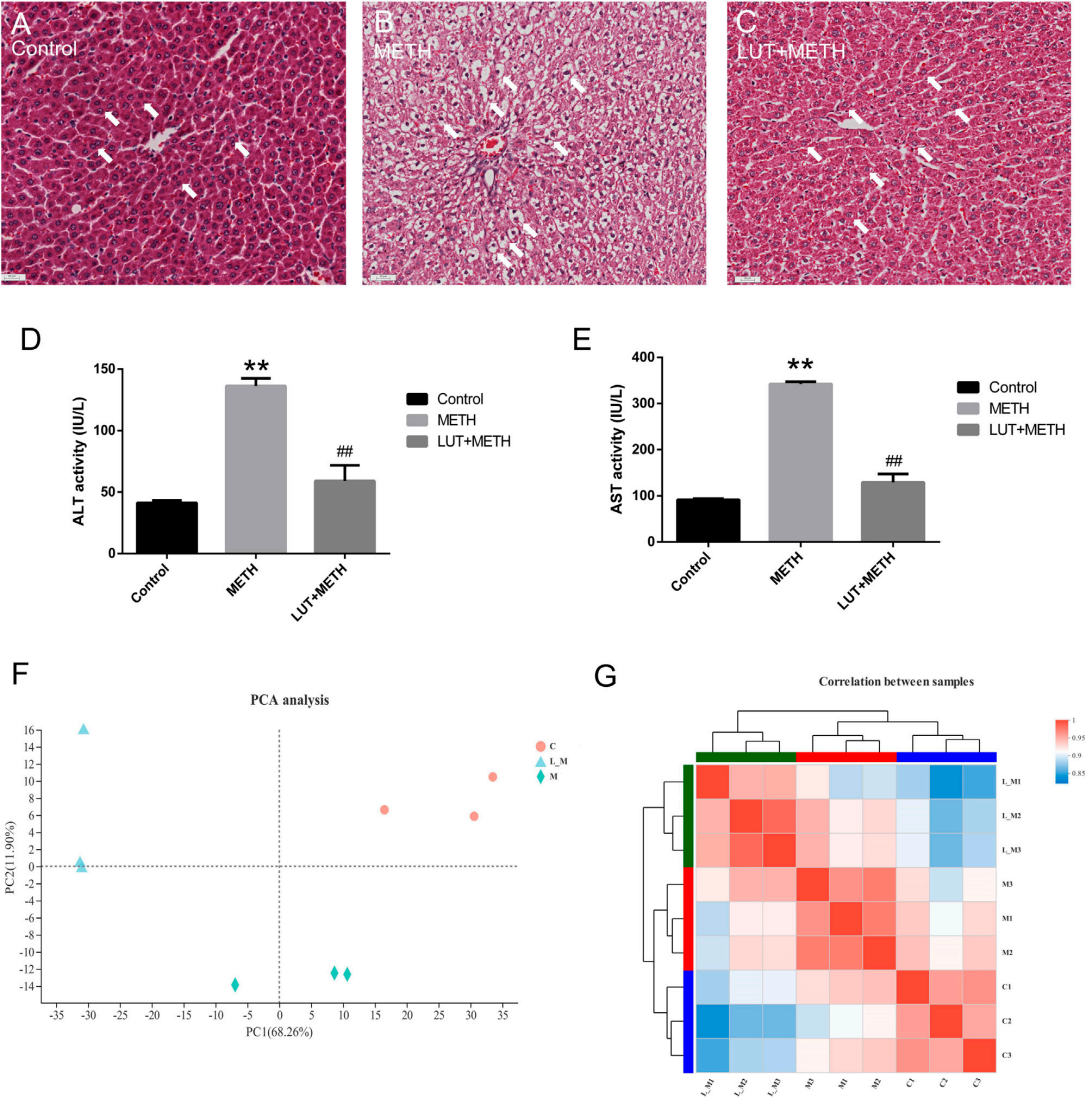
Histopathological and biochemical assessment of liver injury. **(A–C)** Compared to the control group, METH-induced serious hepatocyte ballooning. Luteolin + METH group alleviated liver injury in comparison to the METH group. **(D, E)** METH significantly enhanced the level of ALT and AST in plasma relative to the control group. The increasing level was attenuated in the Luteolin + METH group.**p < 0.01, compared with control group, ^##^ *p <* 0.01, compared with METH group (mean ± SEM). Principal correlation analysis and correlation analysis among groups. **(F)** According to the principal component, intra-group samples were clustered respectively, and then, three groups were separated distinctly. L_M, luteolin + METH. (G) Correlation analysis among samples. The tree presents the correlation among samples, whereas the color displays the coefficient of correlation.

Histopathological analysis showed that 15 mg/kg METH significantly damaged the microstructure of hepatic cells by mediating extensively hepatocyte ballooning in the test group compared to the control group (Figures 2A,B). Compared with the METH group, the pathological changes were alleviated by the pretreatment of luteolin (Figure 2C).

Biochemical indexes, ALT and AST, can mirror the function and the damage degree of the liver. Therefore, these indexes were also examined in this study. METH significantly elevated the plasma level of ALT and AST. These increased indexes were attenuated by the pretreatment of luteolin (Figures 2D,E).

### Screening of the Potentially Protective Mechanisms of Luteolin

#### Principal Component (PCA) and Correlation Analysis

The PCA analysis can simplify the RNA-seq reads and directly reflect the principal components. The distance of spots represented the similarity of principal components. Samples of the control group (red circles), METH group (green rhombuses), and luteolin pretreated group (blue triangles) were clustered separately (Figure 2F), which meant that the higher the difference between groups the lower variation intra-group. Correlation analysis produced similar results. Intra-group samples were clustered and shown a higher correlation compared to samples from other groups (Figure 2G).

#### Screening of DEGs

**FIGURE 3:**
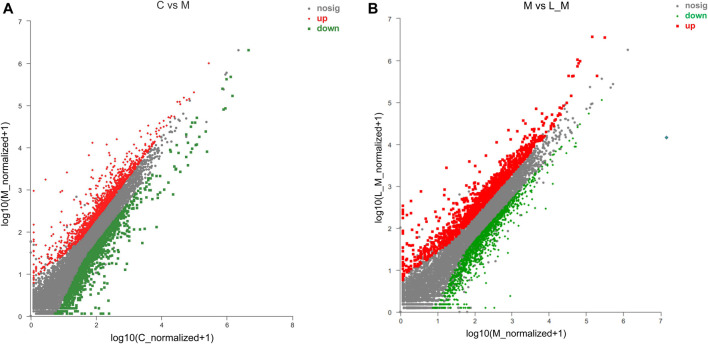
Identification of DEGs using the Scatter plot method. **(A)** METH treatment induced the DEGs, relative to the control group; **(B)** compared to the METH group, luteolin pretreatment produced DEGs (the fold change ≥2 or ≤0.5, *p*-value < 0.05). The red plots represent the up-regulated DEGs, whereas the down-regulated DEGs are represented by green plots.

METH significantly up-regulated 873 DEGs and down-regulated 986 DEGs in comparison to the control group (Figure 3A). Luteolin pretreatment also induced 899 DEGs up-regulation and 978 DEGs down-regulation in the test group compared to the METH group (Figure 3B). Among the METH-induced DEGs, 497 DEGs could be regulated through luteolin treatment (314 up-regulated and 183 down-regulated DEGs).

#### Functional Annotation of DEGs

**FIGURE 4:**
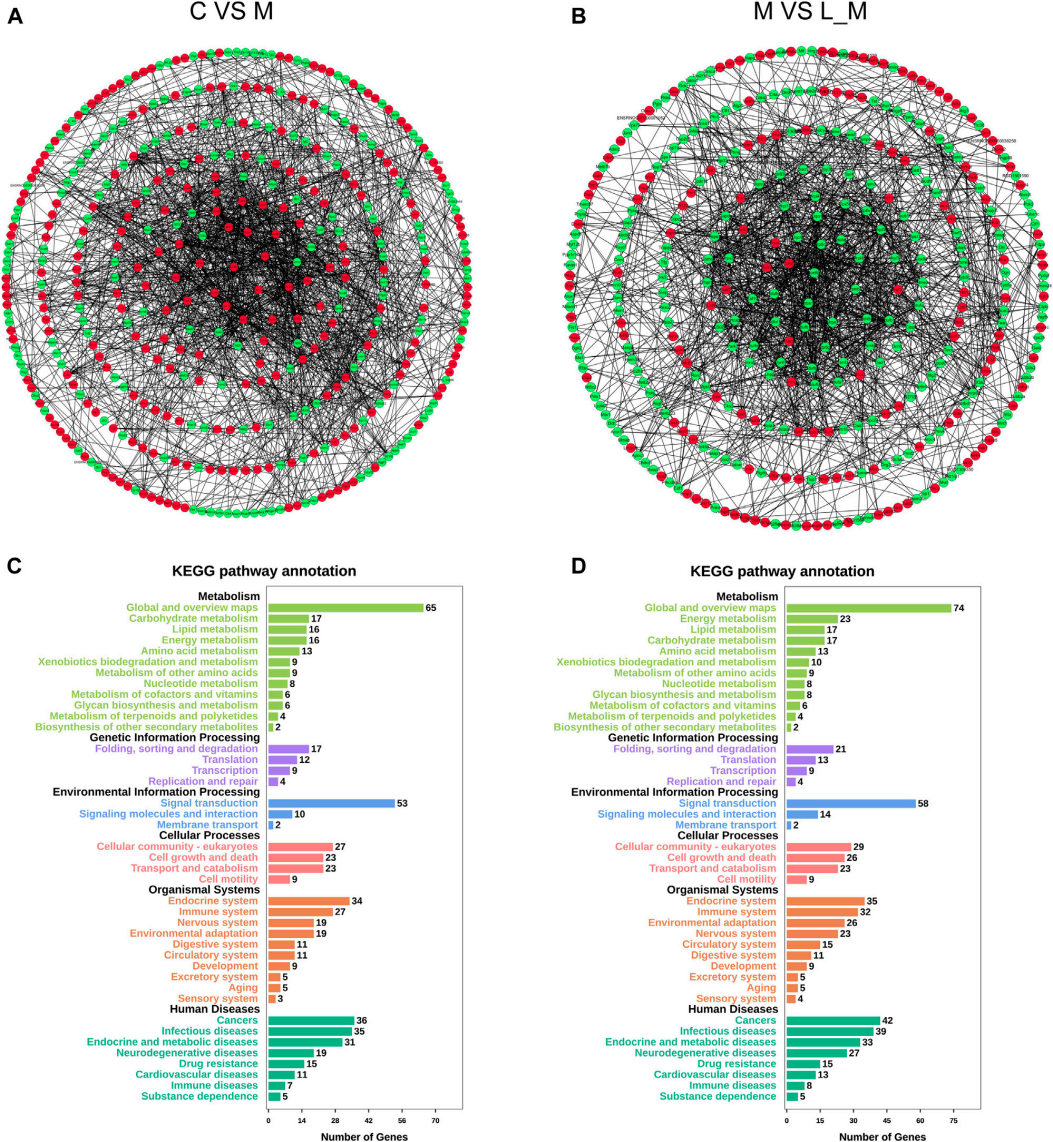
PPI and KEGG analysis of DEGs in the METH and luteolin pretreated group **(A, B)** PPI analysis revealed the direct and indirect connections among DEGs. Up-regulated DEGs were represented by red, and down-regulated DEGs were remarked with green **(C, D)** *X*-axis; the number of the enriched DEGs, *Y*-axis; the second level term of gene ontology.

The interactions among these DEGs were analyzed through PPI analysis to investigate the underlying mechanisms of METH hepatotoxicity and luteolin’s protective effects. Genes with direct or indirect connections were linked, whereas the unrelated or unrecognized genes were eliminated automatically. The more the connections between proteins, the closer they were to the center of the circle, which suggested the strong relationships among the identified DEGs (Figures 4A,B).

The KEGG analysis further provided the clues of the pathways involved. The METH-induced DEGs were mainly enriched into 11 pathways, whereas the DEGs which could be regulated by luteolin were enriched into 16 pathways (Tables 1,2). Interestingly, the p53 signaling pathway, which has been reported to mediate the toxicity of METH, was enriched in both groups. The DEGs from two groups were further annotated according to the classification of metabolism, genetic information processing, environmental information processing, cellular processes, organismal systems, and human diseases (Figures 4C,D).

**TABLE 1:** Pathway enrichment of the METH group.

Pathway enrichment					
No	Pathway ID	Pathway	Count	%	*p*-value
1	rno04151	Metabolic pathways	54	0.070,537,522	0.001,386,404
2	rno00190	Oxidative phosphorylation	12	0.015,675,005	0.002,143,915
3	rno00510	N-Glycan biosynthesis	7	0.009,143,753	0.002,439,666
4	rno05012	Parkinson’s disease	12	0.015,675,005	0.003,272,982
5	rno01130	Biosynthesis of antibiotics	14	0.018,287,506	0.00778,871
6	rno03050	Proteasome	6	0.007,837,502	0.009,337,467
7	rno05010	Alzheimer’s disease	12	0.015,675,005	0.012,312,085
8	rno04152	AMPK signaling pathway	9	0.011,756,254	0.025,718,304
9	rno04540	Gap junction	7	0.009,143,753	0.035,091,577
10	rno04115	p53 signaling pathway	6	0.007,837,502	0.044,351,924
11	rno05206	MicroRNAs in cancer	9	0.011,756,254	0.045,162,063

**TABLE 2:** Pathway enrichment of luteolin pre-treated group.

Pathway enrichment					
NO	Pathway ID	Pathway	Count	%	*p*-value
1	rno01100	Metabolic pathways	62	0.086,741,189	9.24344E-06
2	rno00190	Oxidative phosphorylation	16	0.022,384,823	1.03828E-05
3	rno05012	Parkinson’s disease	16	0.022,384,823	1.99834E-05
4	rno05010	Alzheimer’s disease	16	0.022,384,823	0.00016011
5	rno01130	Biosynthesis of antibiotics	17	0.023,783,875	0.000387,545
6	rno05016	Huntington’s disease	15	0.020,985,772	0.001,562,905
7	rno04932	Non-alcoholic fatty liver disease (NAFLD)	13	0.018,187,669	0.001,843,786
8	rno03320	PPAR signaling pathway	7	0.00979,336	0.02,007,818
9	rno00480	Glutathione metabolism	6	0.008,394,309	0.022,249,139
10	rno00270	Cysteine and methionine metabolism	5	0.006,995,257	0.024,500,584
11	rno00900	Terpenoid backbone biosynthesis	4	0.005,596,206	0.025,077,535
12	rno04152	AMPK signaling pathway	9	0.012,591,463	0.026,348,516
13	rno04540	Gap junction	7	0.00979,336	0.035,775,325
14	rno03050	Proteasome	5	0.006,995,257	0.041,183,838
15	rno04115	p53 signaling pathway	6	0.008,394,309	0.045,094,341
16	rno05206	MicroRNAs in cancer	9	0.012,591,463	0.046,195,794

### Pretreatment of Luteolin Alleviates METH-Induced Apoptosis via Repressing p53 Pathway

**FIGURE 5:**
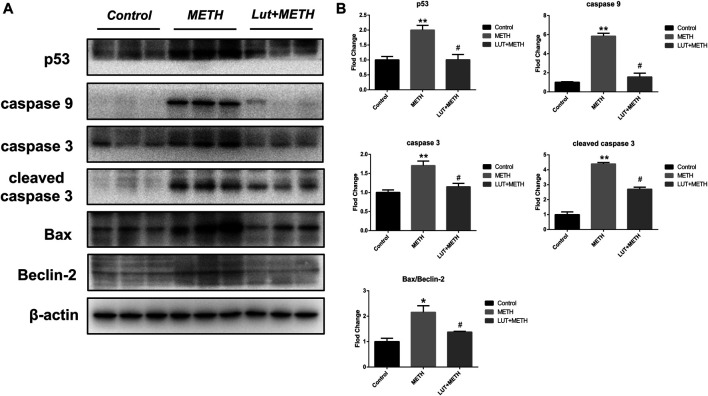
Western blotting analysis for the expression of p53, caspase9, caspase3, cleaved caspase3, Bax, Beclin-2 **(A, B)** Compared to the control group, METH significantly increased the expression level of p53, caspase9, caspase3, cleaved caspase3, Bax, and the normalized ratio of Bax/Beclin-2. These alterations were eliminated by luteolin pretreatment. *p < 0.05*,* **p < 0.01, compared with control group, ^#^ *p <* 0.05, compared with METH group (mean ± SEM).

Results from KEGG analysis show a significant alteration of the p53 pathway in both treatment groups. Therefore, the western blotting analysis was employed to study this pathway. For the test group compared to the control group, METH significantly increased the expression of p53 and its downstream apoptosis-related proteins, including caspase9, caspase3, cleaved caspase3, Bax, and the ratio of Bax/Beclin-2, whereas the up-regulation of these proteins were effectively repressed by luteolin pretreatment (Figures 5A,B).

### Pretreatment of Luteolin Restrains METH-Induced Autophagy and Inflammation

**FIGURE 6:**
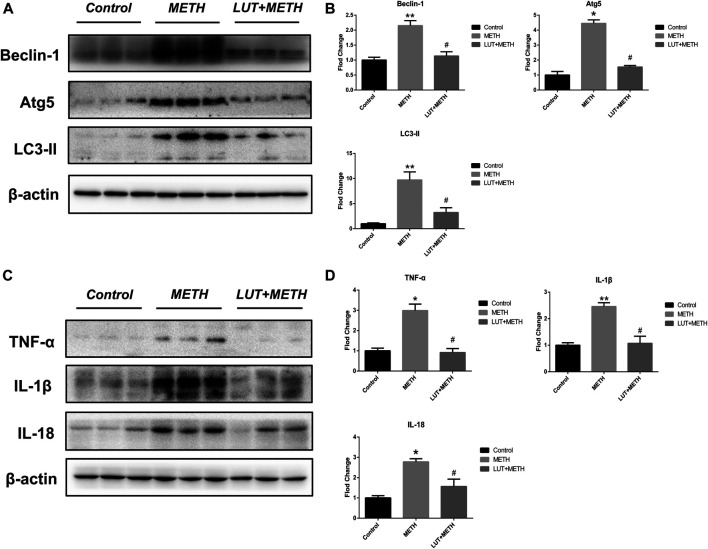
Western blotting analysis for the expression of Beclin-1, Atg5, LC3-II, TNF-α, IL-1β, and IL-18 **(A, B)** Expression of Beclin-1, Atg5, and LC3-II were elevated after METH treatment in comparison with the control group, but these overexpressions were relieved by luteolin treatment **(C, D)** Similarly, METH also up-regulated the level of TNF-α, IL-1β, and IL-18, but these effects were diminished by luteolin. *p < 0.05*,* **p < 0.01, compared with control group, ^#^ *p <* 0.05, compared with METH group (mean ± SEM).

The expression of autophagy-related proteins was also observed given the close relation between autophagy and the p53 signaling pathway. The expression levels of Beclin-1, Atg5, and LC3-II were increased following METH treatment. Luteolin restrained the up-regulation of these proteins (Figures 6A,B).

There was a significant elevation of inflammatory factors compared to the control group; TNF-α, IL-1β, and IL-18 after METH treatment. Luteolin pretreatment also significantly alleviated the METH-induced high expression of these proteins (Figures 6C,D).

### Preatment With Luteolin Fails to Reverse METH-Induced Repression of PI3K/Akt Pathway

**FIGURE 7:**
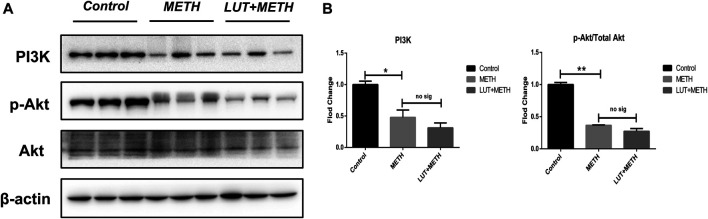
Western blotting analysis for the expression of PI3K, *p*-Akt, and Akt **(A, B)** Relative to the control group, PI3K, *p*-Akt, and the normalized ratio of *p*-Akt/Akt were enhanced following METH treatment, while these effects weren’t attenuated by luteolin pretreatment. *p < 0.05*,* **p < 0.01, compared with control group, no sig, there was no statistical significance, compared with METH group (mean ± SEM).

Luteolin alleviates METH-induced neurotoxicity by modulating PI3K/Akt pathway (Tan et al., 2020). Here, this pathway was studied. There was a significant decline of related proteins following METH treatment: PI3K, Akt phosphorylation (*p*-Akt), and the *p*-Akt/Akt ratio. Interestingly, these low expression proteins weren’t reversed through luteolin pretreatment (Figures 7A,B).

## Discussion

In our previous study, luteolin showed protective effects on METH-induced hepatotoxicity (Qu et al., 2020), though the potential mechanisms were not clear. In this study, we confirmed that luteolin effectively alleviated METH-induced hepatic-pathological changes and decreased biochemical indexes of ALT and AST. The p53 pathway was enriched in both the METH and luteolin pretreated groups via KEGG analysis, based on the RNA-seq data. Downstream, the expression of apoptosis- and autophagy-related proteins were up-regulated following METH treatment, which was attenuated by luteolin pretreatment. Moreover, METH-induced inflammation was also repressed by luteolin. The PI3K/Akt pathway was suppressed after METH treatment, while this effect wasn’t reversed by luteolin. These results suggested that luteolin could protect against METH mediated hepatic toxicity by repressing the p53 pathway.

RNA-sequencing data provided the detailed mechanisms of METH-induced hepatotoxicity and luteolin’s protective effects. According to the KEGG analysis, eight pathways were both enriched into the METH group and luteolin pre-treated group. Among these pathways, metabolic pathways were the most enriched pathways. Conceivably, METH exposure has been reported to induce the serious disfunction of hepatic metabolism (Zhang et al., 2019). As a conserved master regulator of metabolism, the AMPK signaling pathway has been recognized as the potential therapeutic target of hepatic metabolic diseases (Smith et al., 2016; Garcia et al., 2019). In addition, oxidative phosphorylation provides the most ATP for higher animals and the damage of this pathway could induce the energy metabolism disorder (Wilson, 2017), suggesting the potential mechanism of hyperpyrexia after METH treatment (White, 2002). Interestingly, PD- and AD-related pathways were also enriched in liver tissue. This phenomenon strongly hints the pro-neurodegeneration effects of METH (Shin et al., 2017; Keshavarzi et al., 2019). The reverse regulation of these pathways could be involved in the protective effects of luteolin.

Increasing evidence shows that p53 plays a crucial role in METH-induced toxicity. Here, the p53 signaling pathway was also enriched in METH-induced hepatotoxicity. METH has been reported to mediate neural apoptosis by up-regulating p53 (Imam et al., 2001), whereas special deletion effectively alleviated METH-induced neurotoxicity (Hirata and Cadet, 1997; Lu et al., 2017). Meanwhile, Bax (a cell death effector) and the ratio of Bax/Beclin-2 (molecular markers of cell apoptosis) were also up-regulated after METH treatment, although its liberation regulator Beclin-2 wasn’t altered (Ke et al., 2015; Ali et al., 2018). Activated Bax can mediate the apoptosis cascade of aspartate-specific cysteine proteases by increasing caspase9 (Li et al., 1997; Hakem et al., 1998). Consistently, METH-induced increase of Bax also showed the stimulating effects on apoptosis. METH increased the expression of caspase9 and then elevated the level of caspase3 and cleaved caspase3, which played a crucial role in apoptosis (Porter and Jänicke, 1999). Furthermore, the anti-apoptotic bioactivity of luteolin has been confirmed in multiple *in vivo*/*in vitro* models (Zhang et al., 2016; Liu and Meng, 2018). In the current study, luteolin pretreatment effectively repressed METH-mediated stimulation of the p53 pathway. These findings showed that luteolin significantly suppressed METH-induced overexpression of p53 and then, reduced the level of Bax, the ratio of Bax/Beclin-2, caspase9, caspase3, and cleaved caspase3, suggesting that the p53 pathway could play a key role in the protective effects of luteolin.

There is a complex interplay between autophagy and apoptosis. The activation of p53 functionally intertwines with the autophagic pathway (Baehrecke, 2005; Maiuri et al., 2007; White, 2016). In this study, the up-regulation of autophagy-related proteins following METH injections (Beclin-1, Atg5, and LC3-II) was investigated. Autophagy-related 5 (Atg5) is well-known for its proautophagic activation and can stimulate cell death, which is associated with the processing of microtubule-associated protein light chain 3 (LC3), a marker of autophagy (Tanida et al., 2008; Zheng et al., 2019). Activation of Beclin-1 initiates autophagosome formation and is required for Atg5-dependent autophagy (Kang et al., 2011). Luteolin pretreatment effectively alleviated the up-regulation of these proteins, suggesting that luteolin could resist METH-induced autophagy in rat liver, despite that the interaction between apoptosis and autophagy has not been fully illuminated.

Also, the increase of inflammatory factors, TNF-α, IL-1β, and IL-18, was observed after METH treatment, suggesting the activation of hepatic inflammation. Apoptosis/autophagy has a close association with inflammation and plays the role of checkpoint (Messer, 2017). Both apoptosis and autophagy can be triggered by inflammation and cause the process of interaction (Elmore, 2007; Levine and Kroemer, 2008). Interestingly, the findings showed that both apoptosis and autophagy were activated after METH treatment, implying that inflammation could be the origin of these consequences. Moreover, luteolin pretreatment restrained METH-induced hepatic inflammation by decreasing the high level of TNF-α, IL-1β, and IL-18, which was consistent with the anti-inflammatory effects of luteolin (Park and Song, 2019).

It has been confirmed that p53 can subdue the stimulation of the PI3K/Akt pathway, which represses mTOR (Moore et al., 2008; Grinkevich et al., 2017). This reduces mTOR’s negative regulation to apoptosis and autophagy (Feng et al., 2018). Here, the findings also showed the deactivation of the PI3K/Akt pathway after METH administration. The decreasing level of PI3K and the lower *p*-Akt/Akt ratio implies that apoptosis and autophagy could be stimulated by the suppression of the PI3K/Akt pathway, whereas the overexpression of p53 could initiate it. The blocking effect of METH on the PI3K/AKT pathway was also observed in other *in vivo/in vitro* models, which mediated apoptosis and oxidative stress (Lee et al., 2020; Meng et al., 2020). Interestingly, luteolin pretreatment failed to reverse the deactivation of the PI3K/AKT pathway, indicating that the protection of luteolin on METH-induced hepatotoxicity was independent of PI3K/AKT pathway.

**FIGURE 8:**
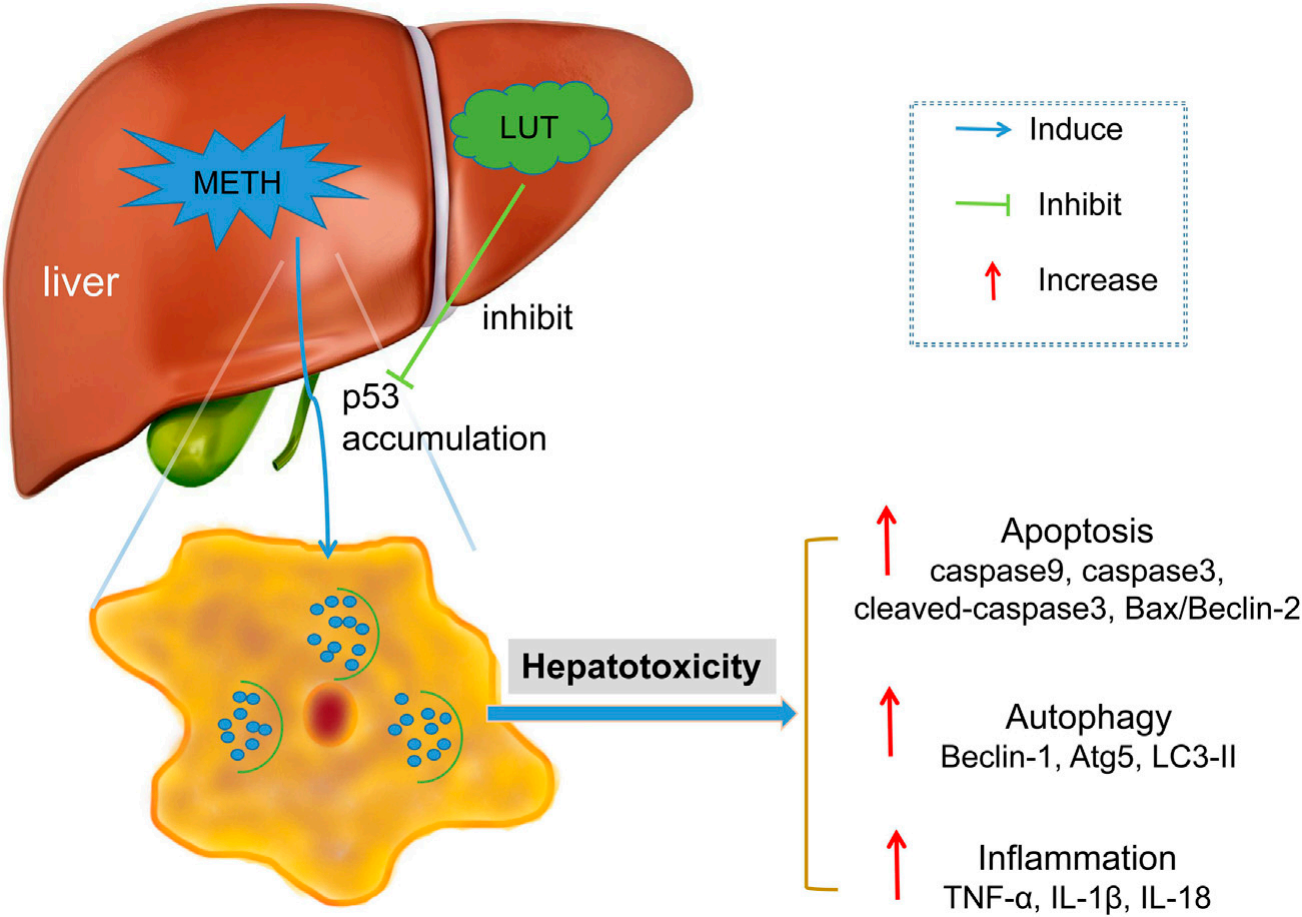
METH stimulated the activation of p53 pathway in hepatic cell, which subsequently mediated apoptosis, autophagy and inflammation. These toxic effects could be attenuated through luteolin pre-treatment via suppressing p53 pathway.

In conclusion, this study showed that the p53 signaling pathway played a key role in METH-induced hepatotoxicity and the protective effects of luteolin. METH stimulates the activation of the p53 pathway, which triggers hepatic apoptosis, autophagy, and inflammation. Notably, these consequences were attenuated by the pretreatment of luteolin by suppressing the p53 pathway (Figure 8). The PI3K/Akt pathway was repressed by METH, though the protection of luteolin on METH-induced hepatotoxicity was independent of PI3K/AKT pathway. This study further investigates the protective mechanisms of luteolin on METH-induced hepatotoxicity, which could serve in the development of treatment drugs.

## Data Availability

The datasets presented in this study can be found in online repositories. The names of the repository/repositories and accession number(s) can be found below: https://www.ncbi.nlm.nih.gov/, PRJNA529763.
